# Supplementary material for: Machine Learning Accelerates Crystallization for Structure Determination
Source: Angew Chem Int Ed Engl. 2026 May 4;65(25):e1218503. doi: 10.1002/anie.1218503 (PMC13266958; doi:10.1002/anie.1218503)

checkCIF/PLATON report

Structure factors have been supplied for datablock(s) lcz009

THIS REPORT IS FOR GUIDANCE ONLY. IF USED AS PART OF A REVIEW PROCEDURE FOR PUBLICATION, IT SHOULD NOT REPLACE THE EXPERTISE OF AN EXPERIENCED CRYSTALLOGRAPHIC REFEREE.

No syntax errors found. CIF dictionary Interpreting this report

Datablock: lcz009

Bond precision:	C-C = 0.0225 A		Wavelength=1.54184
Cell:	a=9.5753(1)	b=14.1910(2)	c=16.1289(2)
	alpha=90	beta=98.210(1)	gamma=90
Temperature:	100 K		

	Calculated	Reported
Volume	2169.18(5)	2169.18(5)
Space group	P 21	P 1 21 1
Hall group	P 2yb	P 2yb
Moiety formula	C35 H31 Ag3 F18 N6 O2	C15 H3 Ag3 F18 N6, 2(C10 H14 O)
Sum formula	C35 H31 Ag3 F18 N6 O2	C35 H31 Ag3 F18 N6 O2
Mr	1233.27	1233.27
Dx,g cm-3	1.888	1.888
Z	2	2
Mu (mm-1)	11.836	11.836
F000	1204.0	1204.0
F000'	1209.42	
h,k,lmax	12,17,20	12,17,20
Nref	9234[4811]	6581
Tmin,Tmax	0.158,0.215	0.414,1.000
Tmin'	0.070	

Correction method= # Reported T Limits: Tmin=0.414 Tmax=1.000
AbsCorr = MULTI-SCAN

Data completeness= 1.37/0.71   Theta(max)= 77.688

R(reflections)= 0.0572(5996)   wR2(reflections)= 0.1557(6581)

S = 1.060   Npar= 577

The following ALERTS were generated. Each ALERT has the format
test-name_ALERT_alert-type_alert-level.
Click on the hyperlinks for more details of the test.

Alert level B

PLAT342_ALERT_3_B Low Bond Precision on C-C Bonds .... 0.0225 Ang.

Author Response: Disordered structure.

PLAT915_ALERT_3_B No Flack x Check Done: Low Friedel Pair Coverage 45 %

Author Response: Due to insufficient data. The compound crystallizes in the chiral space group, but no chiral ligand is used, and the complex is no longer chiral.

Alert level C

PLAT042_ALERT_1_C Calc. and Reported MoietyFormula Strings Differ Please Check
Calc: C35 H31 Ag3 F18 N6 O2
Rep.: C15 H3 Ag3 F18 N6, 2(C10 H14 O)
PLAT090_ALERT_3_C Poor Data / Parameter Ratio (Zmax > 18) .... 7.98 Note
PLAT213_ALERT_2_C Atom F00K has ADP max/min Ratio ..... 3.1 prolat
PLAT234_ALERT_4_C Large Hirshfeld Difference F00B --C00V . 0.22 Ang.
PLAT234_ALERT_4_C Large Hirshfeld Difference F00S --C01D . 0.23 Ang.
PLAT234_ALERT_4_C Large Hirshfeld Difference F010 --C01I . 0.18 Ang.
PLAT234_ALERT_4_C Large Hirshfeld Difference C00I --C01J . 0.19 Ang.
PLAT234_ALERT_4_C Large Hirshfeld Difference C4 --C01L . 0.24 Ang.
PLAT234_ALERT_4_C Large Hirshfeld Difference C01L --C01O . 0.19 Ang.
PLAT241_ALERT_2_C High 'MainMol' Ueq as Compared to Neighbors of C00P Check
PLAT241_ALERT_2_C High 'MainMol' Ueq as Compared to Neighbors of C00X Check
PLAT241_ALERT_2_C High 'MainMol' Ueq as Compared to Neighbors of C019 Check
PLAT241_ALERT_2_C High 'MainMol' Ueq as Compared to Neighbors of C01M Check
PLAT241_ALERT_2_C High 'MainMol' Ueq as Compared to Neighbors of C01O Check
PLAT242_ALERT_2_C Low 'MainMol' Ueq as Compared to Neighbors of C00T Check
PLAT242_ALERT_2_C Low 'MainMol' Ueq as Compared to Neighbors of C014 Check
PLAT242_ALERT_2_C Low 'MainMol' Ueq as Compared to Neighbors of C01L Check
PLAT906_ALERT_3_C Large K Value in the Analysis of Variance ...... 2.207 Check
PLAT911_ALERT_3_C Missing FCF Refl Between Thmin & STh/L= 0.600 18 Report
6 13 0, 1 15 0, 2 15 0, 0 1 1, -2 16 1, 1 16 1,
0 0 2, 6 13 2, 6 14 2, 1 16 2, -3 0 3, 1 0 3,
1 16 4, -4 13 6, 10 0 7, -3 13 12, -9 0 14, -6 7 14,

Alert level G

```
PLAT002_ALERT_2_G Number of Distance or Angle Restraints on AtSite         35 Note
PLAT003_ALERT_2_G Number of Uiso or U(i,j)  Restrained non-H-Atoms         22 Report
PLAT083_ALERT_2_G SHELXL Second Parameter in WGHT  Unusually Large       5.91 Why ?
PLAT171_ALERT_4_G The CIF-Embedded .res File Contains EADP Records          1 Report
PLAT172_ALERT_4_G The CIF-Embedded .res File Contains DFIX Records         22 Report
PLAT177_ALERT_4_G The CIF-Embedded .res File Contains DELU Records          6 Report
PLAT178_ALERT_4_G The CIF-Embedded .res File Contains SIMU Records          5 Report
PLAT186_ALERT_4_G The CIF-Embedded .res File Contains ISOR Records          5 Report
PLAT188_ALERT_3_G A Non-default SIMU Restraint Value has been used     0.0200 Report
PLAT242_ALERT_2_G Low    'MainMol' Ueq as Compared to Neighbors of       C00V Check
PLAT242_ALERT_2_G Low    'MainMol' Ueq as Compared to Neighbors of        C13 Check
PLAT242_ALERT_2_G Low    'MainMol' Ueq as Compared to Neighbors of       C01D Check
PLAT242_ALERT_2_G Low    'MainMol' Ueq as Compared to Neighbors of       C01H Check
PLAT242_ALERT_2_G Low    'MainMol' Ueq as Compared to Neighbors of       C01I Check
PLAT242_ALERT_2_G Low    'MainMol' Ueq as Compared to Neighbors of       C01P Check
PLAT720_ALERT_4_G Number of Unusual/Non-Standard Labels ..........         82 Note
              Ag01    Ag02    Ag03    O004    O005    N006    F007    F009
              N00A    F00B    N00C    F00D    F00E    N00F    F00G    C00H
              C00I    C00J    H00J    F00K    C00L    F00M    N00N    F00O
              C00P    H00A    H00B    F00Q    C00R    H00R    F00S    C00T
              N00U    C00V    F00W    C00X    H00X    C00Y    F00Z    F010
              F011    C012    F013    C014    C015    H015    C016    C017
              H01K    H01L    H01N    C018    H01A    H01B    H01C    C019
              H019    C01A    H01D    H01E    H01F    F01B    C01C    H01O
              C01D    C01E    C01F    C01G    C01H    C01I    C01J    H01J
              C01L    C01M    H01M    C01N    H01G    H01H    H01I    C01O
              H01P    C01P
PLAT791_ALERT_4_G Model has Chirality at C015       (Sohncke SpGr)          S Verify
PLAT791_ALERT_4_G Model has Chirality at C01C       (Sohncke SpGr)          S Verify
PLAT791_ALERT_4_G Model has Chirality at C01J       (Sohncke SpGr)          S Verify
PLAT791_ALERT_4_G Model has Chirality at C01O       (Sohncke SpGr)          S Verify
PLAT860_ALERT_3_G Number of Least-Squares Restraints ............         267 Note
PLAT883_ALERT_1_G Absent Datum for _atom_sites_solution_primary ..     Please Do !
PLAT912_ALERT_4_G Missing # of FCF Reflections Above STh/L=  0.600        187 Note
PLAT913_ALERT_3_G Missing # of Very Strong Reflections in FCF ....          1 Note
                1  0  3,
PLAT933_ALERT_2_G Number of HKL-OMIT Records in Embedded .res File          7 Note
                1 15  0,   1 16  1,   1 16  2,   1 16  4,   1 17  5,   2 15  0,
                6 13  2,
PLAT941_ALERT_3_G Average HKL Measurement Multiplicity ..........         3.0 Low
PLAT969_ALERT_5_G The 'Henn et al.' R-Factor-gap value ..........       2.995 Note
              Predicted wR2: Based on SigI**2  5.20 or SHELX Weight 14.69
PLAT978_ALERT_2_G Number C-C Bonds with Positive Residual Density.          0 Info
```

---

0 **ALERT level A** = Most likely a serious problem - resolve or explain
2 **ALERT level B** = A potentially serious problem, consider carefully
19 **ALERT level C** = Check. Ensure it is not caused by an omission or oversight
28 **ALERT level G** = General information/check it is not something unexpected

2 ALERT type 1 CIF construction/syntax error, inconsistent or missing data
20 ALERT type 2 Indicator that the structure model may be wrong or deficient
9 ALERT type 3 Indicator that the structure quality may be low
17 ALERT type 4 Improvement, methodology, query or suggestion
1 ALERT type 5 Informative message, check

Validation response form

Please find below a validation response form (VRF) that can be filled in and pasted into your CIF.

```
# start Validation Reply Form
_vrf_PLAT042_lcz009
;
PROBLEM: Calc. and Reported MoietyFormula Strings  Differ     Please Check
RESPONSE: ...
;
_vrf_PLAT090_lcz009
;
PROBLEM: Poor Data / Parameter Ratio (Zmax > 18) ........       7.98 Note
RESPONSE: ...
;
_vrf_PLAT213_lcz009
;
PROBLEM: Atom F00K            has ADP max/min Ratio .....         3.1 prolat
RESPONSE: ...
;
_vrf_PLAT234_lcz009
;
PROBLEM: Large Hirshfeld Difference F00B     --C00V     .       0.22 Ang.
RESPONSE: ...
;
_vrf_PLAT241_lcz009
;
PROBLEM: High   'MainMol' Ueq as Compared to Neighbors of       C00P Check
RESPONSE: ...
;
_vrf_PLAT242_lcz009
;
PROBLEM: Low    'MainMol' Ueq as Compared to Neighbors of       C00T Check
RESPONSE: ...
;
_vrf_PLAT906_lcz009
;
PROBLEM: Large K Value in the Analysis of Variance ......      2.207 Check
RESPONSE: ...
;
_vrf_PLAT911_lcz009
;
PROBLEM: Missing FCF Refl Between Thmin & STh/L=    0.600         18 Report
RESPONSE: ...
;
# end Validation Reply Form
```

It is advisable to attempt to resolve as many as possible of the alerts in all categories. Often the minor alerts point to easily fixed oversights, errors and omissions in your CIF or refinement strategy, so attention to these fine details can be worthwhile. It is up to the individual to critically assess their own results and, if necessary, seek expert advice.

PLATON version of 17/09/2025; check.def file version of 17/09/2025

duplicate check

No duplication found

Datablock lcz009 - ellipsoid plot

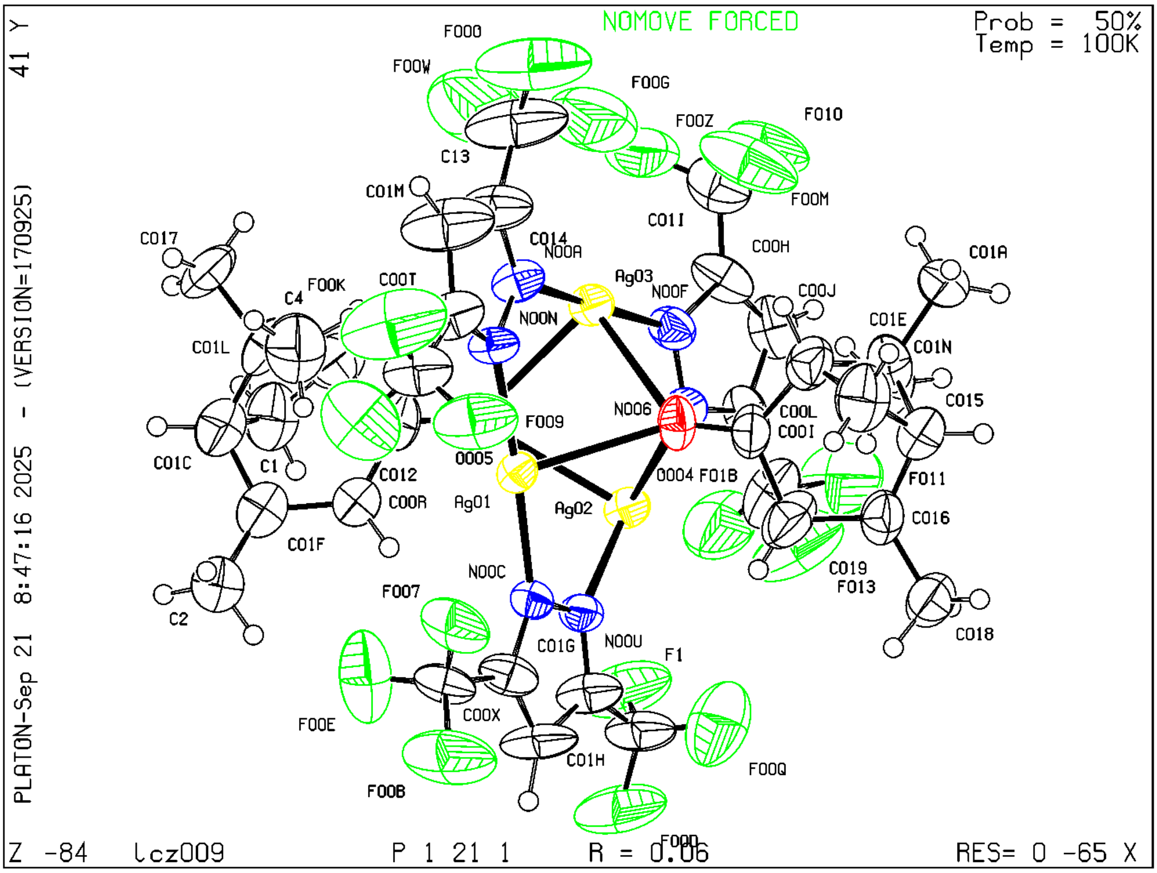

Supplement: Supplementary file 2 — Supporting File 2: anie72462‐sup‐0002‐DataFile.zip. [file ANIE-65-e1218503-s002.zip › supplementary_materials_crystallographic_data submit.zip!/cif_and_checkcif(1-114)/Ag3Pz3_9/Ag3Pz3_9_checkcif.pdf]
